# CD55 Facilitates Immune Evasion by Borrelia crocidurae, an Agent of Relapsing Fever

**DOI:** 10.1128/mbio.01161-22

**Published:** 2022-08-29

**Authors:** Gunjan Arora, Geoffrey E. Lynn, Xiaotian Tang, Connor E. Rosen, Dieuwertje Hoornstra, Andaleeb Sajid, Joppe W. Hovius, Noah W. Palm, Aaron M. Ring, Erol Fikrig

**Affiliations:** a Section of Infectious Diseases, Department of Internal Medicine, Yale University School of Medicine, New Haven, Connecticut, USA; b Department of Immunobiology, Yale University School of Medicine, New Haven, Connecticut, USA; c Amsterdam UMC, University of Amsterdam, Center for Experimental and Molecular Medicine, Amsterdam Infection and Immunity, Amsterdam, Netherlands; McGovern Medical School

**Keywords:** host response, host-pathogen interactions, immunopathogenesis, relapsing fever

## Abstract

Relapsing fever, caused by diverse Borrelia spirochetes, is prevalent in many parts of the world and causes significant morbidity and mortality. To investigate the pathoetiology of relapsing fever, we performed a high-throughput screen of Borrelia-binding host factors using a library of human extracellular and secretory proteins and identified CD55 as a novel host binding partner of Borrelia crocidurae and Borrelia persica, two agents of relapsing fever in Africa and Eurasia. CD55 is present on the surface of erythrocytes, carries the Cromer blood group antigens, and protects cells from complement-mediated lysis. Using flow cytometry, we confirmed that both human and murine CD55 bound to B. crocidurae and B. persica. Given the expression of CD55 on erythrocytes, we investigated the role of CD55 in pathological B. crocidurae-induced erythrocyte aggregation (rosettes), which enables spirochete immune evasion. We showed that rosette formation was partially dependent on host cell CD55 expression. Pharmacologically, soluble recombinant CD55 inhibited erythrocyte rosette formation. Finally, CD55-deficient mice infected with B. crocidurae had a lower pathogen load and elevated proinflammatory cytokine and complement factor C5a levels. In summary, our results indicate that CD55 is a host factor that is manipulated by the causative agents of relapsing fever for immune evasion.

## INTRODUCTION

Borrelia species cause at least two general types of disease in humans: relapsing fever and Lyme borreliosis (1–3). Relapsing fever-associated Borrelia species can cause widespread infection in humans (4–6). Louse-borne Borrelia recurrentis infection was the primary etiologic agent of epidemic relapsing fever in Asia and Europe during the last century (7, 8). The endemic forms of relapsing fever, transmitted by ticks, have been reported in different geographical regions, including North America, Europe, Africa, South America, and Asia (8–12). Relapsing fever is one of the most prevalent bacterial infections in Africa and a significant cause of morbidity in rural areas throughout much of West Africa (13–16).

Tick-borne relapsing fever is characterized by recurrent episodes of systemic symptoms, including headache, myalgias, and bleeding (17). Typically, the first febrile episode lasts for several days, and symptoms recur after afebrile periods of a few days (18–21). The relapsing nature of infection depends on the ability of Borrelia spirochetes to undergo antigenic variation (19). Borrelia miyamotoi is a potential emerging etiologic agent of tick-borne relapsing fever in North America, while Borrelia crocidurae is prevalent in North and West Africa and is emerging in Europe (22, 23). B. crocidurae, which was first isolated from the blood of a musk shrew in Senegal and later identified as the cause of endemic relapsing fever in Western Africa, is a major cause of morbidity and neurologic disease (24, 25). B. crocidurae has been also shown to associate with erythrocytes and generate cell aggregates that disrupt the microcirculation (24, 26, 27). These aggregates create microthrombi in arterioles and subsequently cause myocardial damage (24, 28). In addition, sequestration within aggregates of erythrocytes may allow B. crocidurae spirochetes to avoid damage from the sheer pressure of the blood flow and by contact with immune cells (29).

There is a dearth of information on the host proteins that interact with relapsing fever Borrelia. This information is critical for the development of more effective diagnostics and therapeutics. Molecular and biochemical approaches to identify potential interactions between host immune proteins and spirochete ligands generally require speculation based on putative functionality or bias in the selection of potential immune receptors. We have previously demonstrated that large-scale screening of host-Borrelia interactions with BASEHIT (Bacterial Selection to Elucidate Host-microbe Interactions in high Throughput) effectively overcomes these challenges to identify host factors important in controlling Borrelia pathogenesis *in vivo* (30). Through targeted screening of relapsing fever-causing spirochetes, we determined that CD55, a complement regulator, interacts with B. crocidurae, allowing us to validate its role in erythrocyte aggregation and pathogenesis.

## RESULTS

### Identification of human host factors that interact with spirochetes that cause relapsing fever.

**FIG 1:**
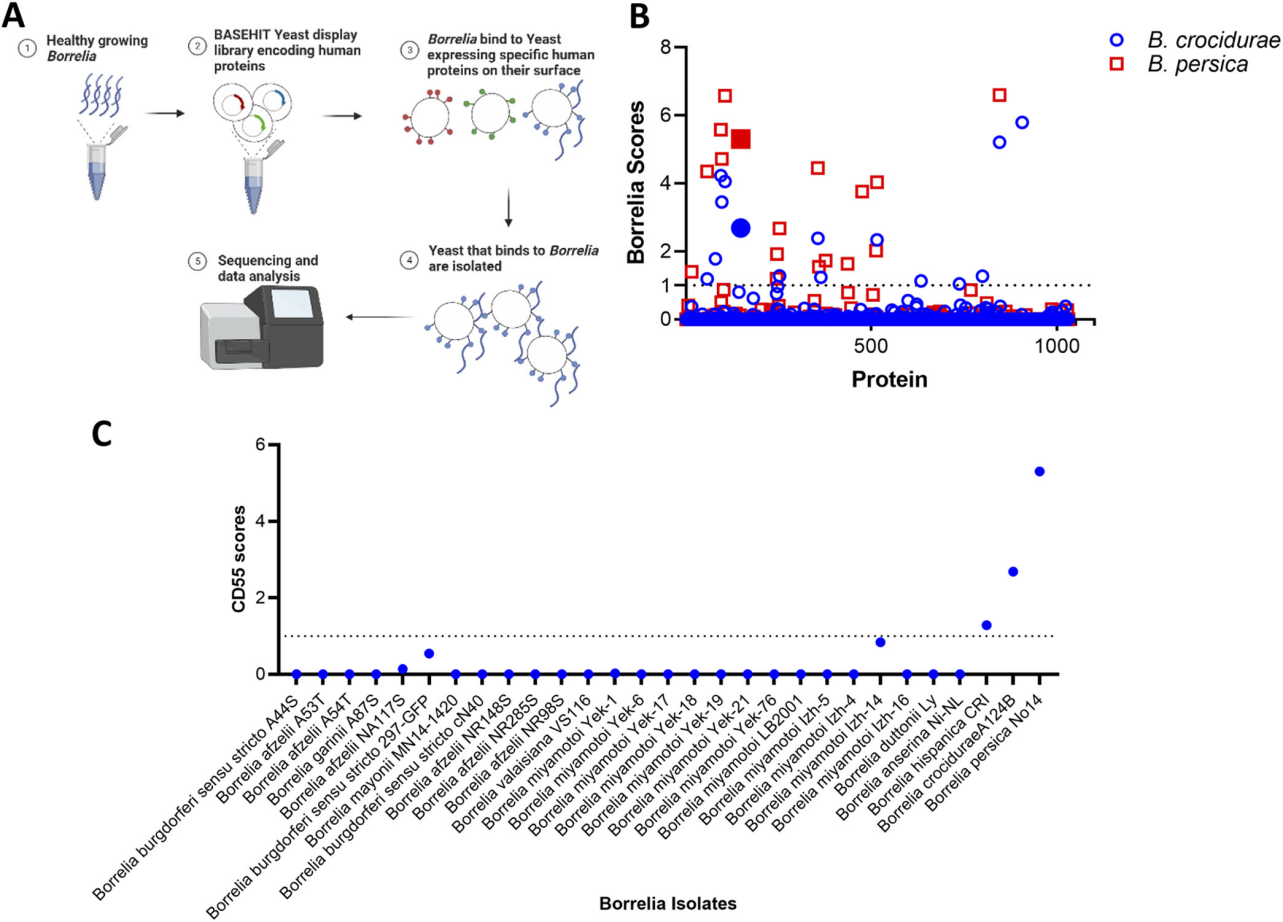
Screening the BASEHIT human exoproteome library to identify host proteins that interact with Borrelia strains that cause relapsing fever. (A) Schematic of yeast display screen: the BASEHIT yeast library, displaying 1,031 human proteins on the yeast surface, was mixed with surface-biotinylated Borrelia isolates. Each human protein is encoded by a unique bar-coded plasmid. Yeast cells binding to Borrelia spirochetes were isolated by magnetic separation using streptavidin microbeads, and next-generation sequencing was used to identify human proteins. (B) Host interactions with B. crocidurae or B. persica grown at 33°C. Each symbol represents one human protein. CD55 is represented by a larger, solid square or circle. The list of proteins that bound to B. crocidurae and/or B. persica is shown in Table S1. The score for each protein is defined as the overall enrichment for that corresponding gene (relative to the unselected library) multiplied by the percentage of barcodes associated with the enriched gene (defined as a log fold change [logFC] of >0). The dotted line reflects a BASEHIT score of 1, and all genes that had higher scores are listed in Table S1. (C) CD55 interactions with different Borrelia species. Samples from 29 *Borrelia* isolates were screened against the host BASEHIT protein library. The *y* axis represents the CD55 score as the overall enrichment for CD55 of each isolate (relative to the unselected library) multiplied by the percentage of barcodes associated with the enriched CD55 (defined as logFC of >0).

Using a recently developed combinatorial screening technology termed BASEHIT (30), we identified specific human proteins that interact with relapsing fever-causing Borrelia species and may therefore be involved in the pathogenesis of or immunity against these microbes. In this approach, surface-biotinylated Borrelia spirochetes are panned against a genetically barcoded Saccharomyces cerevisiae yeast display library of >1,000 human extracellular and secreted proteins. Yeast clones expressing Borrelia-binding proteins are isolated by magnetic separation using streptavidin microbeads and identified by next-generation sequencing of their specific barcode sequences (Fig. 1A).

10.1128/mbio.01161-22.8 TABLE S1 Top hits from yeast display screen for B. crocidurae in order of descending enrichment scores. (A) CD55 is marked in blue (B. crocidurae). (B) Top hits from yeast display screen for *B. persica* in order of descending enrichment scores. CD55 is marked in red (*B. persica*). Download Table S1, PDF file, 0.06 MB. Copyright © 2022 Arora et al. 2022 Arora et al. https://creativecommons.org/licenses/by/4.0/ This content is distributed under the terms of the Creative Commons Attribution 4.0 International license.

Analysis of the results revealed that human CD55 bound to B. crocidurae and Borrelia persica, two spirochetes that cause relapsing fever, and exceeded a stringent significance threshold (Fig. 1B and C; Table S1 in the supplemental material). CD55 is present on the erythrocyte surface and harbors the Cromer blood group antigens (31, 32). CD55 also functions as an endogenous complement inhibitor (33). As blood-borne pathogens, it is imperative for relapsing fever spirochetes to find ways to outwit complement activity (21). Thus, this interaction was of significant interest because it suggested a potential linkage of these Borrelia species with erythrocytes and mechanisms for escape from complement-mediated destruction and other components of the immune system.

### CD55 binds to B. crocidurae and B. persica.

**FIG 2:**
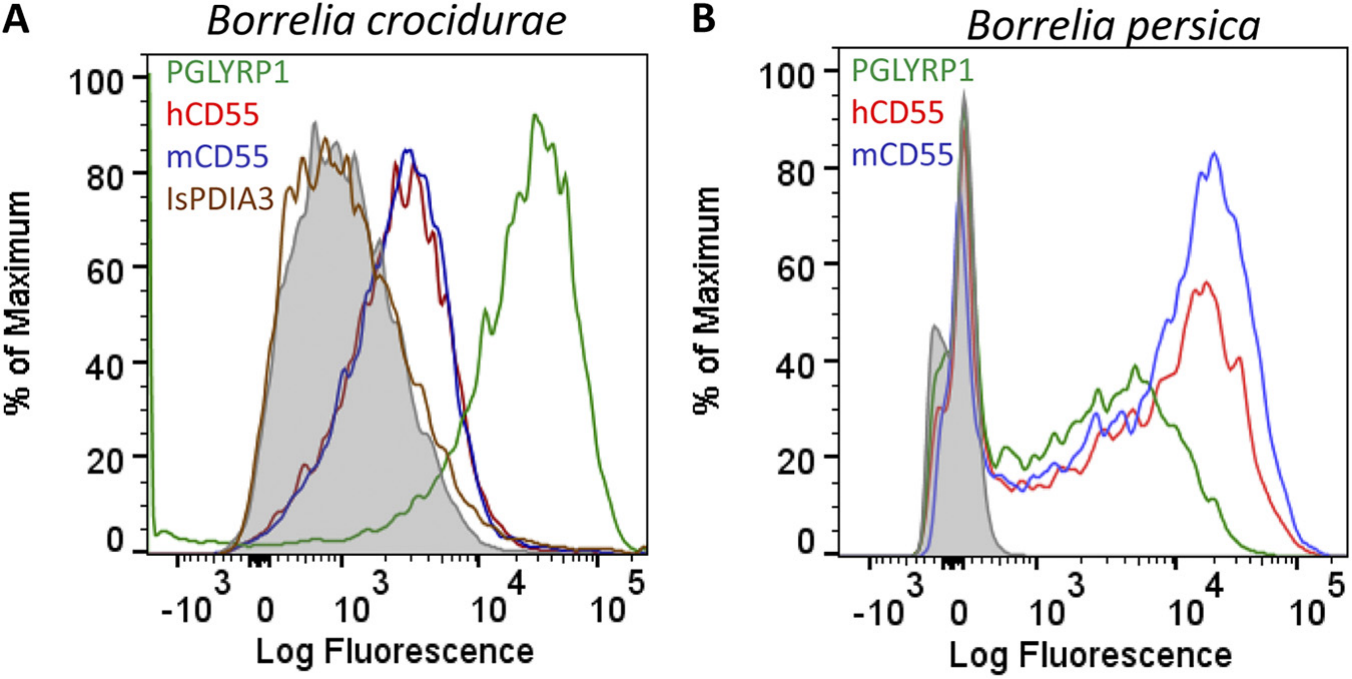
CD55 binds B. crocidurae and B. persica. (A and B) Binding of human and mouse CD55 to B. crocidurae (A) and B. persica (B). (A) B. crocidurae cultures were grown to a density of 10^5^ CFU/mL and incubated with recombinant CD55-His_8_ (50 μg/mL). PGLYRP1 has previously been shown to bind spirochetes that cause Lyme borreliosis and relapsing fever (30) and was used as a positive control. B. crocidurae’s binding to recombinant protein was measured by flow cytometry using a secondary Alexa Fluor 488 (AF488)-conjugated anti-His_6_ monoclonal antibody. Overlay histograms show protein binding to B. crocidurae spirochetes identified using the secondary antibody. Binding of recombinant tick protein IsPDIA3-His_8_ (50 μg/mL) to B. crocidurae was used as a negative control. Background binding of AF488-conjugated anti-His_6_ monoclonal antibody alone with B. crocidurae is shown by the gray-shaded region. Results from one of two representative experiments are shown here. (B) B. persica cultures were grown to a density of 10^5^ CFU/mL and incubated with recombinant CD55-His_8_ (50 μg/mL). B. persica’s binding to recombinant CD55-His_8_ was measured using a secondary AF488-conjugated anti-His_6_ monoclonal antibody.

To determine whether CD55 directly binds to B. crocidurae, we performed flow cytometry-based binding assays with healthy spirochetes grown *in vitro*. Since human CD55 shares 47.21% sequence identity with its mouse orthologue (Fig. S2), we examined whether murine CD55 also recognizes Borrelia spirochetes. As shown by the results in Fig. 2A, both murine and human CD55 bound to a majority of B. crocidurae spirochetes, while the secondary antibody alone showed weak reactivity. As a positive control, we used recombinant human PGLYRP1 (peptidoglycan recognition protein 1), an antimicrobial protein that has been shown to interact with Borrelia species (Fig. 2) (30). As a negative control, we used a poly His-tagged tick protein, IsPDIA3 (Ixodes scapularis protein disulfide isomerase A3) (34), which was also expressed and purified from mammalian cells in the same manner as CD55 and PGLYRP1. To further visualize the interaction between CD55 and B. crocidurae, we performed an immunofluorescence assay with purified CD55-His_8_. This assay further confirmed that CD55 binds to B. crocidurae (Fig. S2A). We also performed flow cytometry-based binding assays with B. persica, B. miyamotoi, or B. duttonii spirochetes and CD55 (Fig. 2B; Fig. S2B and C). As suggested by the BASEHIT screen, CD55 only recognized B. persica among these species. Similar to the results for B. crocidurae, human or mouse CD55 also showed binding to B. persica. Collectively, these results confirmed the interaction of B. crocidurae and B. persica with CD55.

10.1128/mbio.01161-22.1 FIG S1 Sequence alignment of human and mouse CD55. Multiple-sequence alignment of human CD55 (Uniprot identifier P08174) and mouse CD55 (Uniprot identifier Q61475) by using Clustal Omega. Download FIG S1, TIF file, 0.2 MB. Copyright © 2022 Arora et al. 2022 Arora et al. https://creativecommons.org/licenses/by/4.0/ This content is distributed under the terms of the Creative Commons Attribution 4.0 International license.

10.1128/mbio.01161-22.2 FIG S2 CD55 binding with B. crocidurae, B. miyamotoi, and B. duttonii. (A) Binding of CD55 to B. crocidurae visualized by immunofluorescence. B. crocidurae cultures were incubated with recombinant CD55-His_8_ (50 μg/mL) or PGLYRP1 (50 μg/mL) in green. B. crocidurae binding to recombinant protein was measured using anti-His mouse IgG and goat anti-mouse IgG AF488 monoclonal antibody. (B) B. miyamotoi *IZH16* cultures were grown to a density of 10^5^ CFU/mL and incubated with recombinant CD55-His_8_ (50 μg/mL) (human CD55 in red, mouse CD55 in blue). B. miyamotoi binding to recombinant CD55-His_8_ was measured using a secondary AF488-conjugated anti-His_6_ monoclonal antibody. (C) *B. duttonii* cultures were grown to a density of 10^5^ CFU/mL and incubated with recombinant CD55-His_8_ (50 μg/mL) (mouse CD55 in blue) or another poly-His-tagged protein, IsPDIA3 (brown). *B. duttonii* binding to recombinant CD55-His_8_ was measured using a secondary AF488-conjugated anti-His_6_ monoclonal antibody. Download FIG S2, TIF file, 0.2 MB. Copyright © 2022 Arora et al. 2022 Arora et al. https://creativecommons.org/licenses/by/4.0/ This content is distributed under the terms of the Creative Commons Attribution 4.0 International license.

### CD55 binds to a protein ligand on the surface of B. crocidurae.

**FIG 3:**
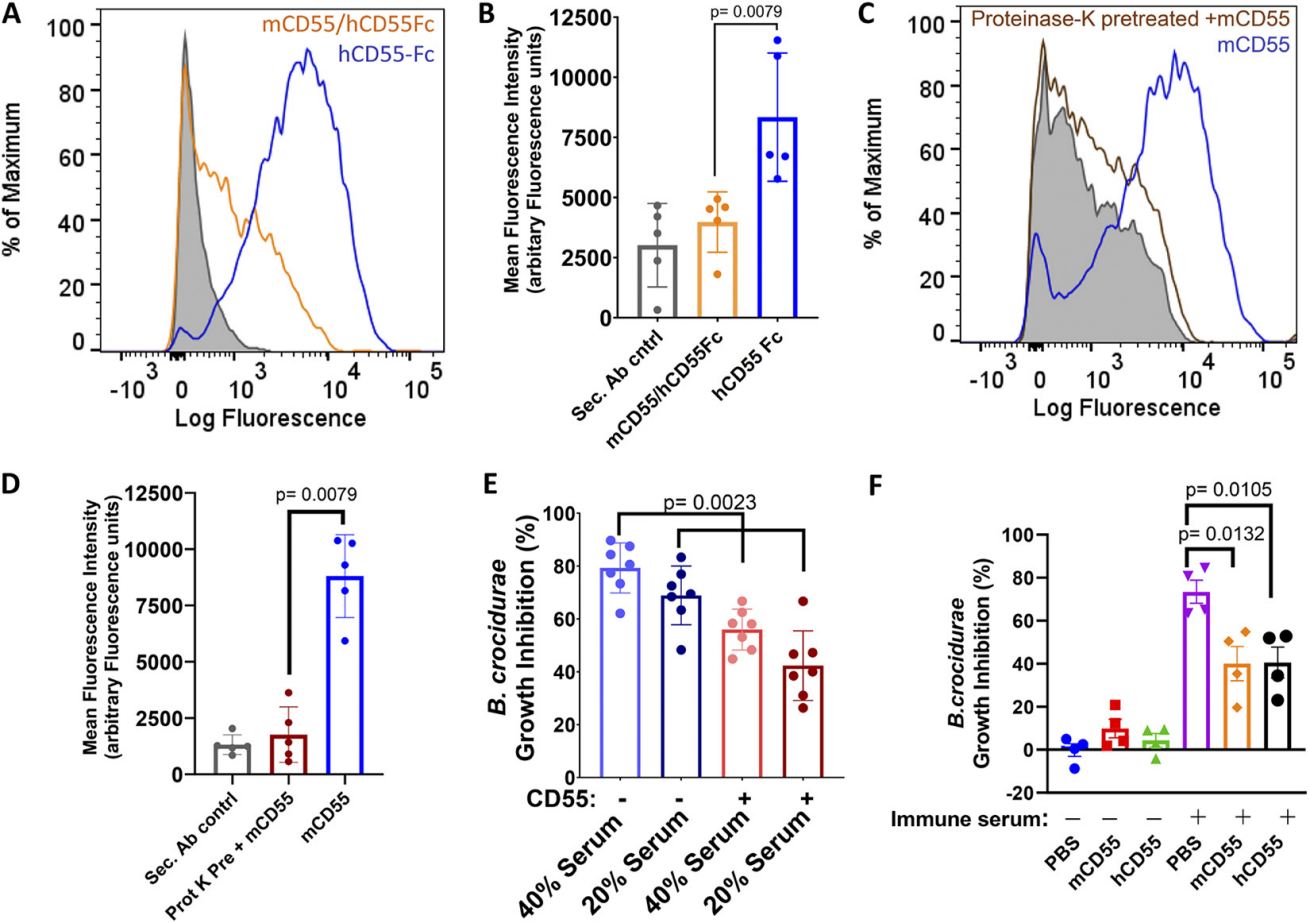
CD55 binding to B. crocidurae and its impact on complement inhibition. (A and B) Binding of human and mouse CD55 to B. crocidurae. (A) B. crocidurae cultures were grown to a density of 10^5^ CFU/mL and incubated with recombinant human CD55-Fc (50 μg/mL) alone or in the presence of mouse CD55-His_8_ (100 μg/mL). B. crocidurae’s binding to human CD55-Fc was measured by flow cytometry using a human CD55 monoclonal antibody (MAB2009; R&D systems) and goat anti-mouse Alexa Fluor 488-conjugated secondary antibody (Thermo Fisher Scientific) (1:1,000). The results from one of five representative experiments are shown here. (B) Data from five independent experiments are shown here. The *y* axis represents arbitrary fluorescence units (mean fluorescence intensity of AF488) from the B. crocidurae spirochete population. Data were acquired on a BD LSRII flow cytometer and analyzed by FlowJo software. The bars represent mean values ± standard deviations (SD), and *P* values were determined by the Student *t* test (Mann-Whitney test of the data for B. crocidurae incubated with hCD55 Fc versus mCD55/hCD55Fc [*P* = 0.0079]). (C and D) Binding of mouse CD55 to proteinase K-treated or untreated B. crocidurae spirochetes. (C) B. crocidurae cultures were grown to a density of 10^5^ CFU/mL and incubated in the presence or absence of proteinase K (0.2 mg/mL) at 37°C for 10 min. Subsequently, the proteinase K activity was quenched using a Roche cOmplete proteinase inhibitor cocktail and spirochetes were washed with PBS thrice. Borrelia spirochetes were incubated with recombinant mouse CD55-His_8_ (50 μg/mL). B. crocidurae’s binding to mouse CD55 was measured by flow cytometry using a secondary AF488-conjugated anti-His_6_ monoclonal antibody. Background binding of AF488-conjugated anti-His_6_ monoclonal antibody alone with B. crocidurae is shown by the gray-shaded region. The results from one of five representative experiments are shown here. (D) Data from five independent experiments are plotted here. The *y* axis represents the mean fluorescence intensities of AF488 from B. crocidurae. Statistical significance was assessed using the nonparametric Student *t* test (Mann-Whitney test of the data for proteinase K-pretreated B. crocidurae incubated with mCD55 versus untreated B. crocidurae incubated with mCD55 [*P* = 0.0079]). (E) Complement-mediated killing of B. crocidurae in the presence or absence of recombinant human CD55. Human CD55 (100 μg/mL) was incubated with B. crocidurae for 2 h in the presence or absence of immune serum from mice that were infected 30 days previously with B. crocidurae. Viability was assessed by observing spirochete movement under dark-field microscopy. The growth inhibition of B. crocidurae was calculated based on the growth of untreated B. crocidurae. The bars represent mean values ± SD, and *P* values were determined by the Student *t* test (Mann-Whitney test of the data for 20% serum versus 20% serum plus hCD55 [*P* = 0.0023] or 40% serum versus 40% serum plus hCD55 [*P* = 0.0023]). Data from seven independent experiments are shown here. (F) Complement-mediated killing of B. crocidurae in the presence or absence of recombinant mouse or human CD55. Human or mouse CD55-His_8_ (100 μg/mL) was incubated with B. crocidurae for 24 h in the presence or absence of 20% serum from mice that were infected 30 days previously with B. crocidurae. Viability was assessed using the Bac-Titer Glo assay. The growth inhibition of B. crocidurae was calculated based on the growth of B. crocidurae incubated with PBS alone. Statistical significance was assessed using the Student *t* test (PBS versus mCD55 [*P* = 0.0132] or PBS versus hCD55 [*P* = 0.0105]). Data from four independent experiments performed in triplicates are shown here.

Since CD55 is conserved in humans and mice and both have complement inhibitory activity (35), we tested whether murine CD55 could competitively inhibit the binding of Fc-tagged human CD55 to B. crocidurae. As shown by the results in Fig. 3A and B, the binding between Fc-tagged human CD55 and B. crocidurae was reduced with the addition of murine CD55-His_8_, suggesting that both proteins bind to the same ligand on the spirochete surface (Fig. 3A and B). To further investigate the nature of the B. crocidurae ligand that interacts with CD55, we performed flow cytometry-based binding assays with protease-treated B. crocidurae spirochetes. Spirochetes treated with proteinase K showed reduced ability to bind to mouse CD55 (Fig. 3C and D). Overall, these results indicate that both human and mouse CD55 bind the same ligand on the B. crocidurae surface.

### CD55 interferes in complement activity against B. crocidurae.

CD55 is known to inhibit complement activity (35–38). To assess this role of CD55 and its effect on binding with the spirochetes, cultured B. crocidurae spirochetes were incubated with immune sera from mice previously infected with B. crocidurae in the presence or absence of recombinant CD55. The borreliacidal activity of mouse complement was first assessed by observing B. crocidurae viability using a dark-field microscope (Fig. 3E). The spirochetes were incubated with either 40% or 20% mouse serum in the presence of human CD55 (100 μg/mL), and Borrelia viability was assessed after 2 h. The borreliacidal activity of immune serum was also assessed by the BacTiter-Glo microbial cell viability assay, which has been performed routinely for live Borrelia estimation. The immune sera had borreliacidal activity that was inhibited by CD55 (Fig. 3F), demonstrating that soluble CD55 can inhibit the complement-mediated killing of Borrelia crocidurae *in vitro*.

### CD55 is involved in B. crocidurae-induced rosette formation.

**FIG 4:**
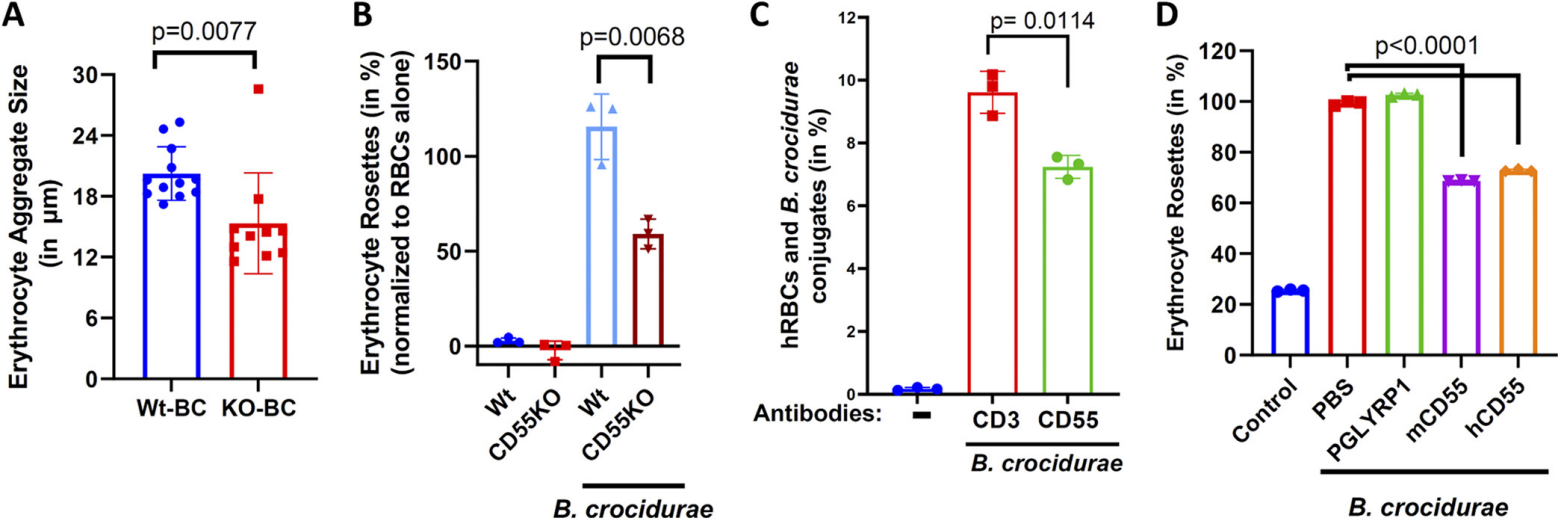
B. crocidurae-induced rosette formation is inhibited by CD55. (A) B. crocidurae interactions with erythrocytes from WT and CD55 KO mice were compared by microscopy. Erythrocytes from WT and CD55 KO mice were labeled with the fluorescent dye PKH67. The labeled RBCs were incubated with 10^5^ B. crocidurae spirochetes in a 96-well, flat-bottom plate at 37°C. The resulting rosettes and aggregates were observed by fluorescence microscopy, and aggregate sizes were measured (Fig. S4). (B) B. crocidurae interactions with erythrocytes from WT and CD55 KO mice were compared. Erythrocytes from WT and CD55 KO mice were incubated with 10^6^ B. crocidurae spirochetes in 0.2-mL PCR tubes at 37°C as described before (39). Hemoglobin from erythrocytes that interacted with B. crocidurae was quantified using the QuantiChrom hemoglobin assay kit. Data from three independent experiments performed in triplicates are presented. (C) B. crocidurae’s interaction with human RBCs in the presence of anti-CD55 neutralizing antibody (R&D Systems) was measured. Human RBCs were preincubated with either anti-human CD55 neutralizing antibody or another antibody (anti-CD3 antibody) as an isotype control for 20 min. The RBCs were incubated with 10^6^ B. crocidurae spirochetes. The interactions between human RBCs and B. crocidurae spirochetes were measured by flow cytometry. Data from three independent experiments are presented. (D) B. crocidurae’s interactions with RBCs in the presence of recombinant CD55 or PGLYRP1 were compared. RBCs from C57BL/6 mice were incubated with 10^6^ B. crocidurae spirochetes at 37°C in the presence or absence of recombinant human (h) or mouse (m) CD55 (100 μg/mL). Hemoglobin from RBCs that interacted with B. crocidurae was quantified.

Relapsing fever spirochetes, including B. crocidurae, are known to form rosettes with erythrocytes, which contributes to the pathogenesis of the relapsing fever and enables spirochetes to evade the host immune system (27). The interaction of relapsing fever-causing Borrelia spp. with erythrocytes is dependent on glycosylation on the surface of erythrocytes (39). However, a specific interaction between relapsing fever spirochetes and an antigen on the erythrocytes has not been demonstrated. CD55 is a glycosylated antigen that is abundantly present on the erythrocyte surface (40, 41). We compared the erythrocyte rosettes induced by B. crocidurae using erythrocytes collected from C57BL/6 wild-type (WT) and CD55 knockout (KO) mice (38). Compared to the results using the erythrocytes isolated from WT mice, B. crocidurae induced fewer rosettes in the presence of erythrocytes from CD55 KO mice (Fig. S3). Furthermore, when the erythrocyte aggregate sizes were compared, erythrocyte aggregates from CD55 KO mice were ~25% smaller than those formed from WT erythrocytes (Fig. 4A; Fig. S4).

10.1128/mbio.01161-22.3 FIG S3 Erythrocyte aggregation in the presence of B. crocidurae as seen under a fluorescence microscope. PKH67-stained RBCs (green) from C57BL/6 and CD55 KO mice were incubated with B. crocidurae, and aggregate numbers were compared. Download FIG S3, TIF file, 0.2 MB. Copyright © 2022 Arora et al. 2022 Arora et al. https://creativecommons.org/licenses/by/4.0/ This content is distributed under the terms of the Creative Commons Attribution 4.0 International license.

10.1128/mbio.01161-22.4 FIG S4 Erythrocyte aggregation in the presence of B. crocidurae as seen under a fluorescence microscope. PKH67-stained RBCs (green) from C57BL/6 and CD55 KO mice were incubated with B. crocidurae, and aggregate sizes were measured. Download FIG S4, TIF file, 1.0 MB. Copyright © 2022 Arora et al. 2022 Arora et al. https://creativecommons.org/licenses/by/4.0/ This content is distributed under the terms of the Creative Commons Attribution 4.0 International license.

To further characterize the effect of CD55 on B. crocidurae-induced rosette formation, rosettes were quantified by endpoint lysis of red blood cells (RBCs) (39). These results confirmed that B. crocidurae forms fewer rosettes with erythrocytes in the absence of CD55 (Fig. 4B). Furthermore, we examined whether B. crocidurae interacted with CD55 present on human erythrocytes. To block CD55 on human RBCs, we preincubated human erythrocytes with a CD55-blocking monoclonal antibody. As a control, we used a CD3 antibody that does not bind erythrocyte antigens. The conjugates between B. crocidurae and human erythrocytes were quantified by flow cytometry. B. crocidurae interacted with human erythrocytes and formed rosettes, while such rosettes were reduced in the presence of anti-CD55 neutralizing antibodies (Fig. 4C). Overall, these results show that B. crocidurae interacts with CD55 present on human or mouse erythrocytes.

Based on these results, we hypothesized that saturating the CD55 binding ligand on B. crocidurae could affect its ability to form rosettes with erythrocytes. To assess this further, B. crocidurae spirochetes were preincubated with either human or mouse recombinant CD55 protein and added to erythrocytes from WT mice. The samples were incubated at 37°C for 20 min before microscopic examination. In the presence of B. crocidurae, RBCs formed visible rosettes, while in the absence of B. crocidurae, erythrocyte aggregates were not observed. When B. crocidurae spirochetes were spiked with recombinant CD55, smaller and fewer erythrocyte aggregates were visible (Fig. 4D; Fig. S5). Rosette formation did not decrease in the presence of an unrelated Borrelia-binding protein, PGLYRP1. Studies have demonstrated the importance of rosette formation in the pathogenesis of B. crocidurae infection (24, 27, 29). These results show that CD55 is a key host protein that is involved in B. crocidurae’s interactions with erythrocytes and evasion of host complement.

10.1128/mbio.01161-22.5 FIG S5 Erythrocyte aggregate formation was observed in the presence of recombinant murine or human CD55 or PGLYRP1. Erythrocytes from C57BL/6 mice were incubated with recombinant human (h) or murine (m) CD55 or PGLYRP1 for 30 minutes before B. crocidurae spirochetes were added. Erythrocyte aggregation was observed under the microscope. Download FIG S5, TIF file, 0.6 MB. Copyright © 2022 Arora et al. 2022 Arora et al. https://creativecommons.org/licenses/by/4.0/ This content is distributed under the terms of the Creative Commons Attribution 4.0 International license.

### CD55-deficient mice show reduced B. crocidurae burden.

**FIG 5:**
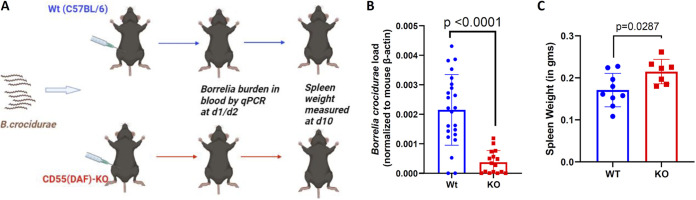
Borrelia burdens and cytokine profiles in CD55 KO mice. (A) B. crocidurae spirochetes were inoculated into WT (C57BL/6) or CD55 KO mice to assess spirochete infection and immune response. The burdens of Borrelia spirochetes in murine blood at 1 and 2 days after infection were determined. The spleen size was determined at 10 days postinfection. (B) Wild-type C57BL/6 and CD55 knockout (KO) mice (*n* = 6 per group minimum) were infected with 10^5^ B. crocidurae spirochetes by intraperitoneal injection. The B. crocidurae burdens in the blood at 2 days postinfection were assessed by qPCR using the Borrelia-specific flagellin subgroup B gene (*flaB*) normalized to mouse *β-actin*. The results from three independent experiments are shown here. (C) The extent of splenomegaly was expressed as splenic weights at 10 days postinfection in Wt and CD55KO mice infected with B. crocidurae. Each data point represents the value for an individual animal. The bars represent mean values ± SD, and *P* values were determined by the Student *t* test.

To understand the physiological significance of the CD55-B. crocidurae interaction, we compared the outcomes of B. crocidurae infection in WT and CD55 KO mice (Fig. 5A). WT and CD55 KO mice were inoculated with 1 × 10^5^ spirochetes. The B. crocidurae burden in the blood was assessed at different days postinfection (dpi) by quantitative PCR (qPCR) using a B. crocidurae *flaB*-specific probe. CD55 KO mice had a significantly lower spirochete burden at 2 dpi (Fig. 5B), suggesting a role for CD55 during the early phase of infection. CD55 KO mice also exhibited splenomegaly compared to the WT group at 10 dpi (Fig. 5C). Furthermore, when blood from infected WT and CD55 KO mice was microscopically examined, B. crocidurae interactions with erythrocytes were more evident in WT mice than in CD55 KO mice (Movies S1 and S2).

10.1128/mbio.01161-22.9 MOVIE S1 Video analysis of B. crocidurae interactions with RBCs from WT (C57BL/6) mouse. Blood was collected at day 7 postinfection, and interactions were visualized by dark-field microscopy. Download Movie S1, AVI file, 16.5 MB. Copyright © 2022 Arora et al. 2022 Arora et al. https://creativecommons.org/licenses/by/4.0/ This content is distributed under the terms of the Creative Commons Attribution 4.0 International license.

10.1128/mbio.01161-22.10 MOVIE S2 Video analysis of B. crocidurae interactions with RBCs from CD55 (DAF) KO mouse. Blood was collected at day 7 postinfection, and interactions were visualized by dark-field microscopy. Download Movie S2, AVI file, 10.3 MB. Copyright © 2022 Arora et al. 2022 Arora et al. https://creativecommons.org/licenses/by/4.0/ This content is distributed under the terms of the Creative Commons Attribution 4.0 International license.

**FIG 6:**
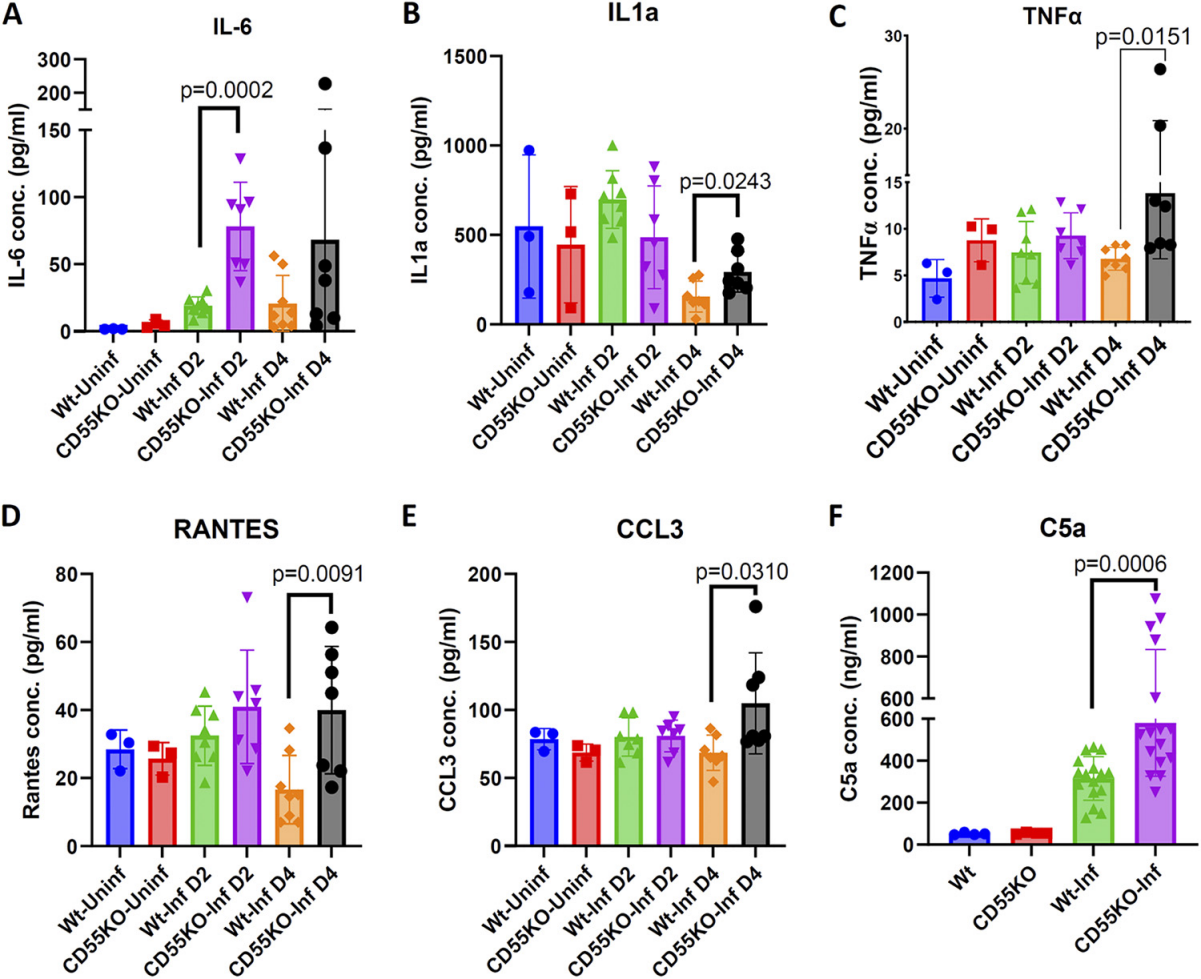
Serological response in CD55 KO mice following B. crocidurae infection. B. crocidurae spirochetes were inoculated into WT (C57BL/6) or CD55 KO mice, and serum cytokine profiles were assessed in both wild-type and CD55 KO mice at day 2 and day 4 postinfection (*n* = 7 in each group) using a mouse cytokine/chemokine 31-plex (MD-31) array. (A to E) Increases in proinflammatory cytokines IL-6 (A), IL-1α (B), and TNF-α (C) and chemokines RANTES/CCL5 (D) and MIP1α/CCL3 (E) were observed in infected CD55 KO mice compared to the levels in parent C57BL/6 mice. Each data point represents the result for an individual animal in the corresponding group. The bars represent mean values ± SD, and *P* values were determined by the Student *t* test. (F) C5a levels in wild-type C57BL/6 (WT) and CD55 KO mice. Four days postinfection, C5a levels were measured by ELISA using serum from infected WT or infected CD55 KO mice. Serum from uninfected mice was used for comparison. All sera were used at a 1:1,000 dilution. Each data point represents the result for an individual animal in the corresponding group. The bars represent mean values ± SD, and *P* values were determined by the Student *t* test.

To further understand the immunopathogenesis of B. crocidurae infection, serum cytokine profiles were assessed in both WT and CD55 KO mice infected with B. crocidurae, using a mouse cytokine/chemokine array panel. Sera from uninfected WT and CD55 KO mice were also probed as baseline controls (Fig. 6A). Increases in the levels of interleukin-6 (IL-6) (day 2), IL-1α (day 4), tumor necrosis factor alpha (TNF-α) (day 4), CCL5 (RANTES) (day 4), and CCL3 (day 4) cytokines were observed in infected CD55 KO mice compared to their levels in WT mice (Fig. 6A to E), while the levels of other representative cytokines were not altered (Fig. S6). CCL3 is involved in both febrile and inflammatory responses (42). A key characteristic feature of relapsing fever infection is increased monocyte and neutrophil numbers. IL-1α and IL-6 are produced in blood by myeloid cells, particularly monocytes (43–45). Interestingly, IL-6 regulates neutrophil trafficking during acute inflammation (46). Our results suggest that the interactions of CD55 with B. crocidurae may also influence the host neutrophil and monocyte response.

10.1128/mbio.01161-22.6 FIG S6 Soluble effectors in WT and CD55 KO mice after B. crocidurae infection. The changes in the levels (pg/mL) of G-CSF, GM-CSF, M-CSF, MIP-1β, IL-1β, CXCL-9, eotaxin, KC, and IL-2 in infected CD55 KO and C57BL/6 mice were not statistically significant. Each data point represents an individual animal in the corresponding group. The bars represent mean values ± SD. Download FIG S6, TIF file, 0.2 MB. Copyright © 2022 Arora et al. 2022 Arora et al. https://creativecommons.org/licenses/by/4.0/ This content is distributed under the terms of the Creative Commons Attribution 4.0 International license.

CD55 is known to control complement activity, including through inhibition of the C5 convertase, and soluble C5a (a cleaved component of complement C5 that signals through the G protein-coupled receptor C5AR) can also induce IL-6 production. To assess whether CD55-mediated control of IL-6 was related to an increase in C5a production, we compared the C5a levels in the serum of WT and CD55 KO mice at 4 dpi. Our results indicated that CD55 KO mice had higher C5a levels at 4 dpi (Fig. 6F). These findings suggest that in the absence of CD55, mice may limit infection by increasing complement and cytokine responses.

### CD55-associated pathways are linked to B. crocidurae pathogenesis.

**FIG 7:**
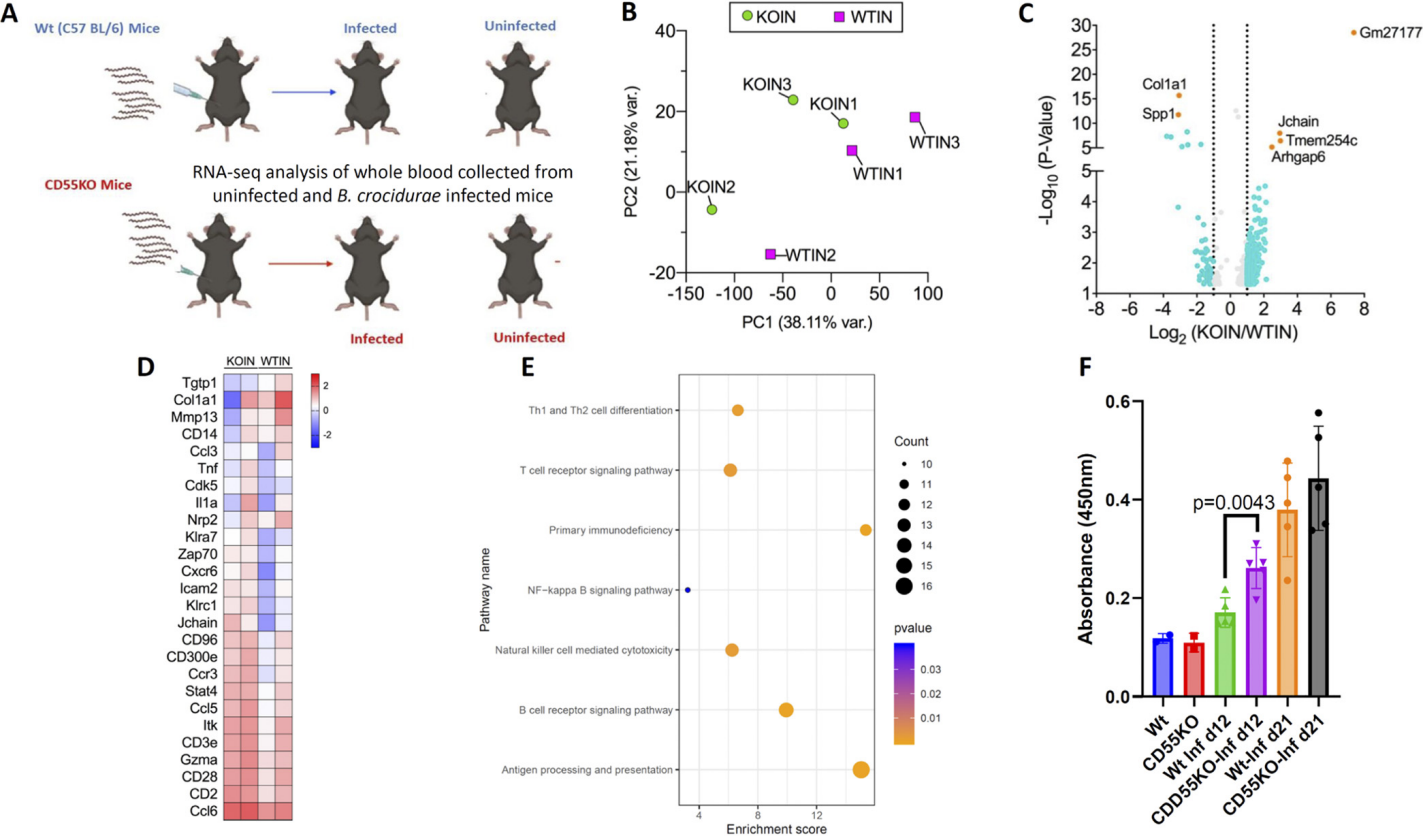
Gene expression analysis by RNA sequencing reveals key genetic signatures following B. crocidurae infection. (A) Transcriptome analysis was done with WT (C57BL/6) or CD55 KO mice to assess genetic signatures following relapsing fever infection. Mice were infected with 10^5^ B. crocidurae spirochetes, and blood was collected at 2 dpi. The results for CD55 KO and C57BL/6 WT mice were compared. (B) Principal-component analysis (PCA) of transcriptome data from murine whole-blood samples. Each dot represents the data for one mouse. KOIN, B. crocidurae-infected CD55 KO mice; WTIN, B. crocidurae-infected C57BL/6 WT mice. (C) Differentially expressed genes. The *x* axis shows the log_2_-transformed fold changes in CD55 KO mice relative to the expression levels in WT mice at 2 days postinfection with B. crocidurae. (D) Heat map of the expression of selected genes in KOIN and WTIN. (E) Signaling pathways involved in the response to B. crocidurae infection in KOIN and WTIN were identified by KEGG pathway enrichment analysis. The key immune pathways enriched included T cell receptor and B cell receptor signaling, natural killer cell-mediated cytotoxicity, NF-κB signaling, antigen processing and presentation, and primary immunodeficiency. (F) B. crocidurae-specific IgM levels in uninfected wild-type C57BL/6 (WT) and CD55 KO mice were compared with those in the infected animals at two time points (12 days and 21 days postinfection). Whole-cell lysate of B. crocidurae was used to coat the wells of a microtiter plate, and serum from uninfected WT, infected WT, uninfected CD55 KO, or infected CD55 KO mice was used at a 1:200 dilution. The binding was measured using a secondary HRP-conjugated anti-mouse IgM. Each data point represents the result for an individual animal in the corresponding group. The bars represent mean values ± SD, and *P* values were determined using the Student *t* test.

To understand the molecular pathways in CD55 KO mice associated with resistance to B. crocidurae infection, we compared the whole-blood transcriptomes of WT and CD55 KO mice that were uninfected or infected with B. crocidurae (Fig. 7A). We found that 320 genes were differentially expressed between uninfected WT and CD55 KO mice, while 906 genes were differentially expressed between B. crocidurae-infected WT and CD55 KO mice. Only 43 common genes were observed between uninfected and infected mice in the absence of CD55, indicating that the 863 genes were altered by the deficiency of CD55 in response to B. crocidurae infection (Fig. 7B to D; Fig. S7). Based on Gene Ontology (GO) functional classification and KEGG pathway analyses of the 863 genes, selected immune response and cytokine signaling pathways were highly enriched in infected CD55 KO mice (Fig. 7E). Of note, the natural killer (NK) cell-mediated cytotoxicity pathway, the B and T cell receptor signaling pathways, and chemokines like CCL5 (which was also validated in the above-described cytokine analysis) (Fig. 6D) were significantly upregulated in CD55 KO mice (Fig. 7D). Transcriptomic analysis also showed that J chain expression was significantly upregulated (fold change [FC] of 7.7) (*P* > 0.0001) in CD55 KO mice that were infected with B. crocidurae. J chain is important for the secretion and polymer formation of IgM and IgA (47). To determine the effect of CD55 deletion on the B. crocidurae-specific IgM response, we measured antibody responses to spirochete antigens via sandwich enzyme-linked immunosorbent assay (ELISA). There was a modest increase in B. crocidurae-specific IgM in the sera obtained from CD55 KO mice compared to the level in sera from WT C57BL/6 mice, collected at 12 dpi (Fig. 7F), while no differences were observed in B. crocidurae-specific IgG levels at 12 dpi (Fig. S7C). Overall, transcriptomic analysis, cytokine measurements, and antibody ELISA results indicated that CD55 deletion affected innate and IgM responses following infection. Taken together, all these data suggest that CD55-mediated immune pathways are critical for B. crocidurae infection and that the relapsing fever agent may influence immune signaling.

10.1128/mbio.01161-22.7 FIG S7 Transcriptome analysis of B. crocidurae-infected mice. (A) Principal component analysis (PCA) of transcriptome data of whole-blood samples from mice. Whole blood was collected from KOUN (CD55 KO uninfected) and WTUN (C57BL/6 uninfected) mice. Each dot represents the data for one mouse. (B) Differentially expressed genes. The *x* axis shows the log_2_-transformed fold changes for CD55 KO mice relative to the expression levels in WT mice. (C) B. crocidurae-specific IgG in mice. Antibody levels in uninfected wild-type C57BL/6 (WT) and CD55 KO mice were compared with those in the infected animals at two time points (12 days and 30 days postinfection). Whole-cell lysate of B. crocidurae was used to coat wells of a microtiter plate, and serum from uninfected WT, infected WT, uninfected CD55 KO, or infected CD55 KO mice was used at a 1:200 dilution. The binding was measured using a secondary HRP-conjugated goat anti-mouse IgG. No significant difference in IgG levels was observed in infected WT compared to infected CD55 KO mice. Each data point represents an the result for an individual animal in the corresponding group. The bars represent mean values ± SD, and *P* values were determined using the Student *t* test. Download FIG S7, TIF file, 0.2 MB. Copyright © 2022 Arora et al. 2022 Arora et al. https://creativecommons.org/licenses/by/4.0/ This content is distributed under the terms of the Creative Commons Attribution 4.0 International license.

## DISCUSSION

B. crocidurae causes relapsing fever infections in Africa, Asia, and Europe (12, 48, 49). In this study, we identified CD55 as a novel host interaction partner with B. crocidurae and B. persica, two etiologic agents of relapsing fever. CD55 was identified using a BASEHIT screening strategy that combines next-generation sequencing with an advanced yeast display library approach. This yeast display library expresses 1,031 human proteins individually on the surface of yeast cells, consisting of secretory and extracellular proteins. Other than CD55, identified hits included REG4 (regenerating family member 4) and selected cytokines and chemokines, such as CCL24 (eotaxin-2), CCL17 and CCL11 (eosinophil chemotactic protein and eotaxin-1), CXCL3 (macrophage inflammatory protein-2-beta [MIP2b]), and IL-29 (interferon lambda [IFN-λ1]), that potentially bound to both B. crocidurae and B. persica. It is also interesting that most of these protein candidates were not top hits in our previous screen with Borrelia burgdorferi. These results suggest that BASEHIT is a powerful approach that is capable of identifying strain- and species-specific host binding partners. We examined the interaction between CD55 and B. crocidurae in greater detail because B. crocidurae is a major cause of human disease, readily infects mice, and is known to bind erythrocytes and generate cell aggregates that disrupt the microcirculation (24, 27, 29).

Our results show that CD55 protects B. crocidurae spirochetes from complement-mediated killing and facilitates interactions with erythrocytes. This effect may be a key strategy adopted by this spirochete to enable its early establishment and dissemination in the blood. CD55 is present on the surface of erythrocytes, where its primary role is to protect erythrocytes from complement-mediated lysis (50, 51). B. crocidurae interacts with erythrocyte surface-localized CD55 and induces the formation of rosettes that allow the spirochete to evade innate immune responses (27). We show that mice lacking CD55 exhibit resistance to B. crocidurae infection, as demonstrated by a lower spirochete burden and enhanced cytokine and chemokine innate immune responses. We determined that CD55-B. crocidurae interactions help in the formation of rosettes, which is a crucial feature of B. crocidurae pathogenesis. Furthermore, protease treatment of B. crocidurae decreases the CD55 binding, indicating that this interaction is likely associated with a protein ligand. Overall, CD55 binding to B. crocidurae is critical for its interaction with erythrocytes and pathogenesis, as well as immune evasion.

CD55 is a known complement regulatory protein, and humans with defects in CD55 develop complement hyperactivation, angiopathic thrombosis, and protein-losing enteropathy (CHAPLE disease), a lethal illness that is due to overactivation of complement and innate immunity (52, 53). We hypothesized that the increased resistance of CD55 KO mice to B. crocidurae infection could be related to increased C5a levels in the serum and antimicrobial defenses. Following B. crocidurae infection, the C5a levels increased more in CD55 KO mice than in control animals. Cytokines like IL-6, IL-1α, and CCL5, produced by monocytes, other myeloid cells, or NK cells, were upregulated. We hypothesize that in the absence of CD55, B. crocidurae infection induces inflammation that then increases C5a, CCL3, and CCL5. Increases in these soluble mediators can result in the recruitment and activation of innate immune cells, including NK cells and monocytes that make TNF-α and IL-6. Finally, RNA sequencing elucidated the specific genetic signature associated with B. crocidurae infection in C57BL/6 (WT) and CD55 KO mice. The activated pathways included chemokine signaling pathways and natural killer cell-mediated cytotoxicity pathways.

The activity of complement is tightly regulated by regulatory proteins (decay-accelerating factor [DAF or CD55], membrane cofactor protein [MCP or CD46], complement receptor 1 [CR1 or CD35], and CD59) to balance the response against pathogens and prevent injury of the host. To evade the complement response, pathogens like B. burgdorferi express many different lipoproteins on their surface that bind complement components and interfere with complement activation (54–57). B. burgdorferi surface antigens bind to soluble complement regulators factor H (FH), factor H-like protein, and C4bp and inhibit the activation of the C1 complex, composed of C1q, C1r, and C1s (55, 58). Similarly, different viruses also adopt strategies to thwart the complement attack (56, 59–61). Parasites like Plasmodium falciparum, Entamoeba histolytica, Trichomonas vaginalis, Trypanosoma cruzi, and Schistosoma spp. also use various strategies to escape complement-mediated killing, including the recruitment of complement regulatory proteins and expression of orthologs of complement regulatory proteins to inhibit complement activity (62, 63).

Decay-accelerating factor (DAF or CD55) was first identified as a complement regulator and is a cell surface receptor that is also present in body fluids in a soluble form (64). In addition to inhibiting the early steps of complement activation, CD55 can also influence the activation of T cells and the natural cytotoxicity of NK cells. CD55 binds to CD97, a leukocyte adhesion marker that is involved in the recruitment, activation, and migration of granulocytes. CD55 deficiency increases CD97 expression on the surface of leukocytes and does not affect receptor signaling (65). B. crocidurae’s interactions with CD55 may also affect its binding to CD97, and the CD55-CD97 axis may also contribute to B. crocidurae pathogenesis *in vivo*. Previous studies have shown that the binding of Escherichia coli adhesin to CD55 leads to the induction of the stress-induced ligand MICA (major histocompatibility complex [MHC] class I-related molecule) on epithelial cells (66). In our studies, we did not see differences in Rae-1 (distantly related to MHC class I proteins) expression in CD55 KO mice following infection with B. crocidurae. The increases in proinflammatory cytokines (IL-6, IL-1α, TNF-α, CCL5 [RANTES], and CCL3) and in complement anaphylatoxin C5a may also influence the Borrelia burden in CD55 KO mice. These results suggest that the influence of CD55 on innate immunity may also contribute to B. crocidurae’s growth in the mice. To delineate the role of CD55 in B. crocidurae pathogenesis, studies can focus on identifying interacting ligand(s) on the B. crocidurae or B. persica surface. Our results also suggest that relapsing fever species can be further divided into CD55 binding and nonbinding species, and studies can explore whether CD55 binding is related to multiphasic antigenic variation (20).

B. crocidurae is the primary cause of endemic relapsing fever in Western Africa (67). The clinical manifestations are related to the ability of B. crocidurae to affect the blood coagulation system (24, 29). Our results demonstrate that CD55 directly influences erythrocyte aggregation and the pathogenesis of B. crocidurae. This interaction shields spirochetes from host immune attack and is associated with an altered host inflammatory response. Our screen also showed that a second relapsing fever spirochete, B. persica, binds to CD55. At present, there is little information about the pathogenesis of B. persica, and further studies will delineate the role of CD55 in B. persica infection and that of other relapsing fever strains, including but not limited to emerging pathogens like B. miyamotoi. Overall, relapsing fever caused by B. crocidurae and other spirochetes remains a major source of morbidity globally (15, 68), and it is important to better understand the mechanisms of infectivity in order to develop new therapeutic strategies for this disease. These findings suggest that CD55 plays an important role in the pathogenesis of B. crocidurae infection.

## MATERIALS AND METHODS

### Ethics statement.

All experiments performed in this study were conducted in accordance with the *Guide for the Care and Use of Laboratory Animals* (69), and efforts were made to reduce animal suffering. Animal experiment protocols were approved by the Institutional Animal Care and Use Committee at Yale University (protocol permit number 07941).

### *Borrelia* culture.

The details of Borrelia culturing can be found elsewhere (30). In brief, 48 isolates of several Borrelia spp. were grown (~10^6^ to 10^7^ cells/mL) at 33°C in Barbour-Stoenner-Kelly H (BSK-H) complete medium (product number B8291; Sigma-Aldrich) with 6% rabbit serum or in MKP medium (30, 70). Borrelia spirochetes were washed with phosphate-buffered saline (PBS) and incubated with 5 μM sulfo-NHS-LC-biotin (catalog number 2326-50; BioVision) at 37°C for 30 min, and glycerol stocks (10% vol/vol in PBS) were made for future use in yeast display screening assays. Low-passage-number (<5 passages) isolates of B. crocidurae, B. persica, Borrelia hispanica, Borrelia miyamotoi, Borrelia duttonii, and Borrelia anserina grown at 33°C in MKP growth medium were used in the study.

### Yeast library screening.

Details of library construction and selection are described elsewhere (30). Briefly, a library of barcoded plasmids containing the extracellular portions of 1,031 human proteins was expressed in Saccharomyces cerevisiae strain JAR300 and maintained in SDO-Ura (synthetic drop-out medium, prepared with 20 g/L glucose according to the manufacturer’s instructions) (D9535; USBiological). Protein synthesis was induced by culturing the library in medium containing 90% galactose and 10% glucose for 24 h at 30°C. Induced yeast cells were harvested and incubated with biotinylated bacteria for 1 h at 4°C. Yeast cells were incubated with streptavidin microparticles (0.29 μm) (catalog number SVM-025-5H; Spherotech) for 1 h at 4°C. Bead-bound yeasts were selected by magnetic separation and subsequently grown in 1 mL SDO-Ura at 30°C. DNA was extracted from yeast cell libraries and amplified and sequenced using Illumina MiSeq and Illumina version 2 MiSeq reagent kits according to the manufacturer’s standard protocols. Enrichment calculations were performed using edgeR (30, 71, 72). The overall enrichment for a gene (relative to the unselected library) was multiplied by the percentage of barcodes associated with the enriched gene (defined as a logFC of >0). The cutoff was selected as a BASEHIT score of 1. This was decided based on our previous study, where nearly all Borrelia species showed interaction with PGLYRP1 at a score of >1 (30). This cutoff score allowed a focus on genes that were highly enriched in the BASEHIT screen and therefore bound more strongly to Borrelia species.

### Gene cloning and expression.

Human and mouse CD55 (human CD55, amino acids 35 to 353, or mouse CD55, amino acids 35 to 362) genes were cloned into pEZT-Dlux, a modified pEZT-BM vector, as described before (30). Protein was purified from the culture supernatant by Ni-nitrilotriacetic acid (NTA) chromatography and desalted into PBS. The human PGLYRP1 (amino acids 22 to 196) gene was also cloned into pEZT-BM and the expressed protein purified as described before (30). Expi293 cells (ThermoFisher) were transfected with CD55 or PGLYRP1 using the ExpiFectamine 293 transfection kit (ThermoFisher). Protein purity was verified by SDS-PAGE. The protein concentration was measured by the absorbance at 280 nm.

### Flow cytometry-based CD55 binding assay.

Low-passage-number B. crocidurae spirochetes were cultured to a density of ~10^6^ to 10^7^ cells/mL, washed two times with PBS, and incubated with recombinant human CD55, mouse CD55, IsPDIA3, or PGLYRP1 (all with an 8×His tag) at 4°C for 1 h. After the incubation period, spirochetes were washed three times and fixed in 2% paraformaldehyde (PFA). Spirochetes were blocked in 1% bovine serum albumin (BSA), probed with anti-6×His monoclonal antibody conjugated to Alexa Fluor 488 (AF488) (catalog number MA1-21315-488; ThermoFisher), and run through a BD LSR II instrument (BD Biosciences). These data were analyzed by FlowJo. For competition assays, B. crocidurae spirochetes were incubated with recombinant human CD55-Fc (50 μg/mL) alone or in the presence of mouse CD55-His_8_ (100 μg/mL). The binding of B. crocidurae to human CD55-Fc was measured using an anti-human CD55 mouse monoclonal antibody (MAB2009; R&D systems) and a goat anti-mouse IgG (H+L) Alexa Fluor 488- conjugated secondary antibody (ThermoFisher Scientific) (1:1,000). For the protease assays, B. crocidurae cultures were grown to a density of 10^5^ CFU/mL and incubated in the presence or absence of proteinase K (0.2 mg/mL) at 37°C for 10 min. Subsequently, the proteinase K activity was quenched using a Roche cOmplete proteinase inhibitor cocktail, and spirochetes were washed with PBS thrice. Borrelia spirochetes were incubated with recombinant mouse CD55-His_8_ (50 μg/mL). The binding of B. crocidurae spirochetes to mouse CD55 was measured by flow cytometry using a secondary AF488-conjugated anti-6×His monoclonal antibody.

### Immunofluorescence assay for analysis of B. crocidurae’s interactions with human and mouse CD55.

Spirochetes were grown to a density of ~10^7^ cells/mL and harvested by centrifugation at 5,000 × *g* for 15 min. Cells were washed twice with PBS containing 2% BSA (PBS-BSA). Spirochetes were incubated with either recombinant human or mouse CD55 or human PGLYRP1 conjugated with a His tag or with a control protein fused with a His tag at 50 μg/mL for 1 h at 4°C. After washing two times with PBS and incubating with an anti-His tag AF488-conjugated secondary antibody (1:50), the samples were incubated for an additional 30 min as described before (73). The spirochetes were washed with PBS and visualized by dark-field microscopy.

### Complement activity against B. crocidurae.

The effect of complement and antibodies on B. crocidurae growth was measured using the microscopy and BacTiter Glo microbial cell viability assay. To measure complement-mediated killing of B. crocidurae in the presence or absence of recombinant CD55, human CD55 (100 μg/mL) was incubated with B. crocidurae for 2 h in the presence or absence of immune serum (40% and 20%) from mice that were infected 30 days previously with B. crocidurae. Viability was assessed by observing spirochete movement under dark-field microscopy as described before (74, 75). The growth inhibition of B. crocidurae was calculated based on the results for untreated B. crocidurae.

The BacTiter Glo microbial cell viability assay provides a method for determining the number of viable Borrelia spirochetes in culture based on quantitation of the ATP present by measuring luminescence. The luminescent signal is proportional to the ATP concentration, thus indicating the number of viable Borrelia spirochetes in the culture. To test the effect of human or mouse CD55 on mouse complement and the growth of B. crocidurae, we incubated 1 × 10^5^ B. crocidurae spirochetes under microaerophilic conditions at 33°C for 24 h in a final volume of 300 μL in the presence of immune serum. Immune serum was collected from mice at day 30 postinfection with B. crocidurae and stored at −80°C in aliquots. The percentage of growth inhibition was calculated using viable spirochetes that were incubated in the absence of serum.

### Quantitative erythrocyte rosetting assay.

Log-phase B. crocidurae spirochetes were harvested by centrifugation at 8,000 × *g* and resuspended to 1 × 10^8^ spirochetes/mL in RPMI containing 10% fetal bovine serum (FBS). B. crocidurae and erythrocytes were preincubated at 37°C for 15 min in different tubes. After preincubation, B. crocidurae spirochetes and erythrocytes were mixed in 96-well, flat-bottom microtiter plates and incubated at 37°C for 30 min. The rosettes were visualized under the EVOS microscope (ThermoFisher). For analyzing the rosette size, the erythrocytes were labeled with the fluorescent dye PKH67 before the assay. The excess dye was quenched using FBS, and erythrocytes were washed with RPMI. The rosette size was calculated using EVOS cell imaging system software.

### Erythrocyte rosetting assay by microscopy.

Log-phase B. crocidurae spirochetes were harvested by centrifugation at 8,000 × *g* and resuspended to 1 × 10^8^ spirochetes/mL in RPMI containing 10% FBS. B. crocidurae spirochetes and erythrocytes were preincubated at 37°C for 15 min in different tubes. After the preincubation, 20 μL of B. crocidurae spirochetes and 40 μL of erythrocytes were mixed in 0.2-mL PCR strip tubes and incubated at 37°C for 15 min. Subsequently, 40 μL of supernatant was removed from each tube and 50 μL of fresh medium was added to the tube. The tube was further incubated for 15 min at 37°C, and subsequently, 50 μL of erythrocytes floating at the top was removed without disturbing the rosettes. At the end of the incubation, 200 μL of water was added to lyse the RBCs, and 50 μL of lysed erythrocyte solution was used to measure hemoglobin, using the QuantiChrom hemoglobin assay kit and measuring absorbance at 405 nm.

### Flow cytometry-based interaction assay.

Human RBCs from a healthy donor were used for these assays. RBCs were washed twice with PBS and subsequently stained with the cell proliferation dye eFluor 670 (ThermoFisher Scientific) at 5 μM for 5 min in PBS at 37°C. Similarly, healthy growing B. crocidurae spirochetes were stained with EvaGreen dye (Biotium) for 5 min in PBS at 37°C. RBCs and spirochetes were then separately washed three times with medium containing serum (RPMI with 10% FBS). RBCs were preincubated in the presence of 5 μg anti-human CD55 antibody (MAB2009; R&D Systems) or anti-CD3 antibody (Biolegend). For interaction assays, spirochetes and RBCs were incubated together for 30 min at 37°C and 5% CO_2_ in a humidified chamber. Flow cytometry was performed on a FACS LSR-II (BD Biosciences), and data analyzed with FlowJo (FlowJo, LLC).

### *In vivo* infection of mice.

Pathogen-free C57BL/6 wild-type (WT) mice (Charles River Laboratories) and CD55 KO mice (C57BL/6 DAF^−/−^) (38) were used for *in vivo* experiments. The CD55 mice were verified by immunostaining of RBCs from C57BL/6 and CD55 KO mice. The RBCs were stained with antibodies against Ter119 and mouse CD55 (Biolegend).

Pathogen-free C57BL/6 WT mice (Charles River Laboratories) and CD55 KO mice (C57BL/6 DAF^−/−^) at 6 to 8 weeks of age were infected intraperitoneally with low-passage-number B. crocidurae (1 × 10^5^ spirochetes). Uninfected mice were used as controls. Blood was collected at different days postinfection to compare the Borrelia burdens. Spleen weights were measured at day 10 postinfection immediately following euthanasia to assess splenomegaly. The protocol for the use of mice was reviewed and approved by the Yale Animal Care and Use Committee.

For video analysis of B. crocidurae interaction with RBCs, blood was collected at day 7 postinfection. The whole blood was immediately diluted in PBS, and interactions were visualized by dark-field microscopy within 1 h of blood draw.

### Quantification of B. crocidurae burden.

B. crocidurae DNA was extracted from whole-blood samples using the DNeasy blood and tissue kit (Qiagen). Quantitative PCR (qPCR) was performed using i*Taq* universal SYBR green supermix (Bio-Rad). For quantitative detection of the B. crocidurae burdens within mouse blood samples, qPCR with DNA was performed using the flagellin subgroup B gene (*flaB*), a marker for B. crocidurae detection. The primers used in the assay were Flab F, GAATTAATCGTGCATCTGAT, and Flab R, CATCCAAATTTCCTTCTGTTG. The mouse *β-actin* gene (30, 73) was used to normalize the amount of DNA in each sample.

### Cytokine profile.

Serum collected from each group of mice (at day 2 postinfection and day 4 postinfection) was sent for cytokine analysis by the 31-plex mouse cytokine/chemokine array (MD-31) performed by Eve Technologies as described before (30). The cytokines represented by this array are eotaxin, granulocyte colony-stimulating factor (G-CSF), granulocyte-macrophage colony-stimulating factor (GM-CSF), IFN-γ, IL-1α, IL-1β, IL-2, IL-3, IL-4, IL-5, IL-6, IL-7, IL-9, IL-10, IL-12 (p40), IL-12 (p70), IL-13, IL-15, IL-17A, IP-10, KC, LIF, LIX, MCP-1, macrophage colony-stimulating factor (M-CSF), MIG, MIP-1α, MIP-1β, MIP-2, RANTES, TNF-α, and vascular endothelial growth factor receptor (VEGF).

### RNA-seq analysis.

Total RNA was extracted from whole blood obtained from mice 2 days after the intraperitoneal infection with B. crocidurae. TRIzol was added to the whole blood, and RNA was isolated according to the manufacturer’s instructions (Qiagen, CA). RNA was submitted for library preparation using TruSeq (Illumina, San Diego, CA, USA) and sequenced using the Illumina HiSeq 2500 by paired-end sequencing at the Yale Center for Genome Analysis (YCGA). All the transcriptome sequencing (RNA-seq) analyses, including alignment, quantitation, normalization, and differential gene expression analyses, were performed using Partek Genomics Flow software (St. Louis, MO, USA). Specifically, RNA-seq data were trimmed and aligned to the mouse genome (mm10) with the associated annotation file using STAR (version 2.7.3a) (76). The aligned reads were quantified by comparison to Ensembl transcripts release 91 using the Partek E/M algorithm (77), and the subsequent steps of gene-level annotation followed by total count normalization were performed. The gene-level data were normalized by dividing the gene counts by the total number of reads, followed by the addition of a small offset (0.0001). Principal-component analysis (PCA) was performed using default parameters for the determination of the component number, with all components contributing equally in Partek Flow. Volcano plot hierarchal clustering was performed on the genes that were differentially expressed across the conditions (*P* < 0.05, fold change of ≥2 for each comparison). Pathway enrichment was also conducted in Partek Flow as described before (78). A gene expression heatmap of the selected genes was further plotted by using ggplot2 and Prism version 8 (GraphPad). The selected immune pathways were further plotted on a bubble diagram by using ggplot2 in R studio.

### Measuring the C5a levels in mouse serum by ELISA.

The mouse serum was collected at day 4 postinfection. For C5a measurements, the sera were diluted 1:1,000. Mouse complement component C5a DuoSet ELISA kits (R&D Systems, Minneapolis, MN, USA) were used according to the manufacturer’s recommendations.

### Antibody titers against B. crocidurae.

IgM and IgG titers against B. crocidurae were detected in mouse sera by ELISA as described above. Briefly, wells were coated with lysate of B. crocidurae, blocked, and incubated with mouse sera diluted in 1% BSA at different titers (1:200 and 1:2,000). After washing, HRP-conjugated goat anti-mouse IgM (1:10,000) (catalog number 62-6840; ThermoFisher) or HRP-conjugated rabbit anti-mouse IgG (1:10,000) (catalog number 61-6520; ThermoFisher) was added. KPL Sureblue tetramethylbenzidine (TMB) 1-component microwell peroxidase substrate was added, and the reaction was stopped with 2 M sulfuric acid. The absorbance of wells was read at 450 nm.

### Statistical analysis.

The analysis of all data was performed with the Student *t* test or analysis of variance (ANOVA) in Prism 8.0 software (GraphPad Software, Inc., San Diego, CA). A *P* value of <0.05 was considered statistically significant.

### Data availability.

The RNA-seq data are available in the Gene Expression Omnibus (GEO) repository at the National Center for Biotechnology Information under the accession number: GSE198510.
