# Supplementary material for: CD55 Facilitates Immune Evasion by Borrelia crocidurae, an Agent of Relapsing Fever
Source: mBio. 2022 Aug 29;13(5):e01161-22. doi: 10.1128/mbio.01161-22 (PMC9600505; doi:10.1128/mbio.01161-22)
Supplement: TABLE S1 [file mbio.01161-22-s0008.pdf]

Supplementary Table

Table 1A

Protein	B. crocidurae
TAC4	5.798394441
REG4	5.208224537
CCL11	4.233209857
CCL28	4.054672908
CCL17	3.456082275
**CD55**	**2.685075101**
FGFBP3	2.388883478
IL29	2.340352319
C1QTNF4	1.781144908
CXCL3	1.280130708

Table 1B

Protein	B. persica
REG4	6.599572098
CCL28	6.578180263
CCL11	5.577914594
**CD55**	**5.304832451**
CCL17	4.723853139
FGFBP3	4.455182
BTC	4.350838994
IL29	4.034356132
IL17C	3.761717863
CXCL3	2.672867628
