# Supplementary figures and images for: CD55 Facilitates Immune Evasion by Borrelia crocidurae, an Agent of Relapsing Fever
Source: mBio. 2022 Aug 29;13(5):e01161-22. doi: 10.1128/mbio.01161-22 (PMC9600505; doi:10.1128/mbio.01161-22)

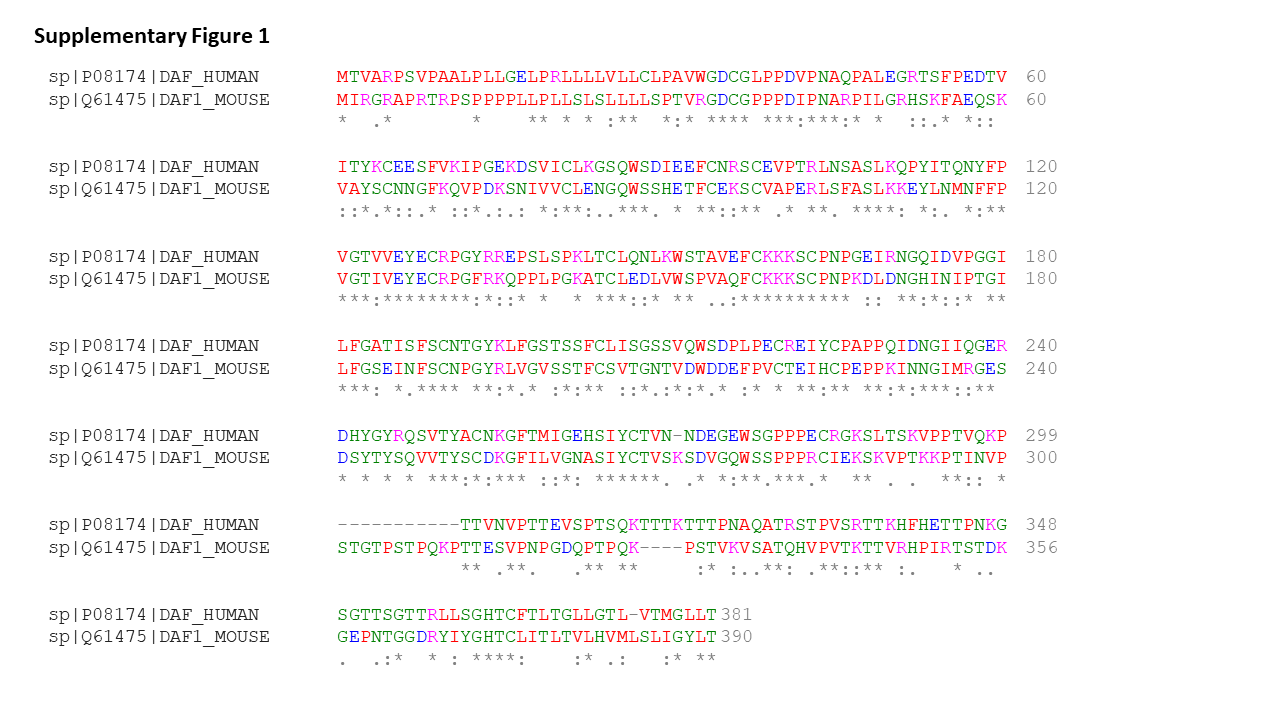

Supplement: FIG S1 [file mbio.01161-22-s0001.tif]

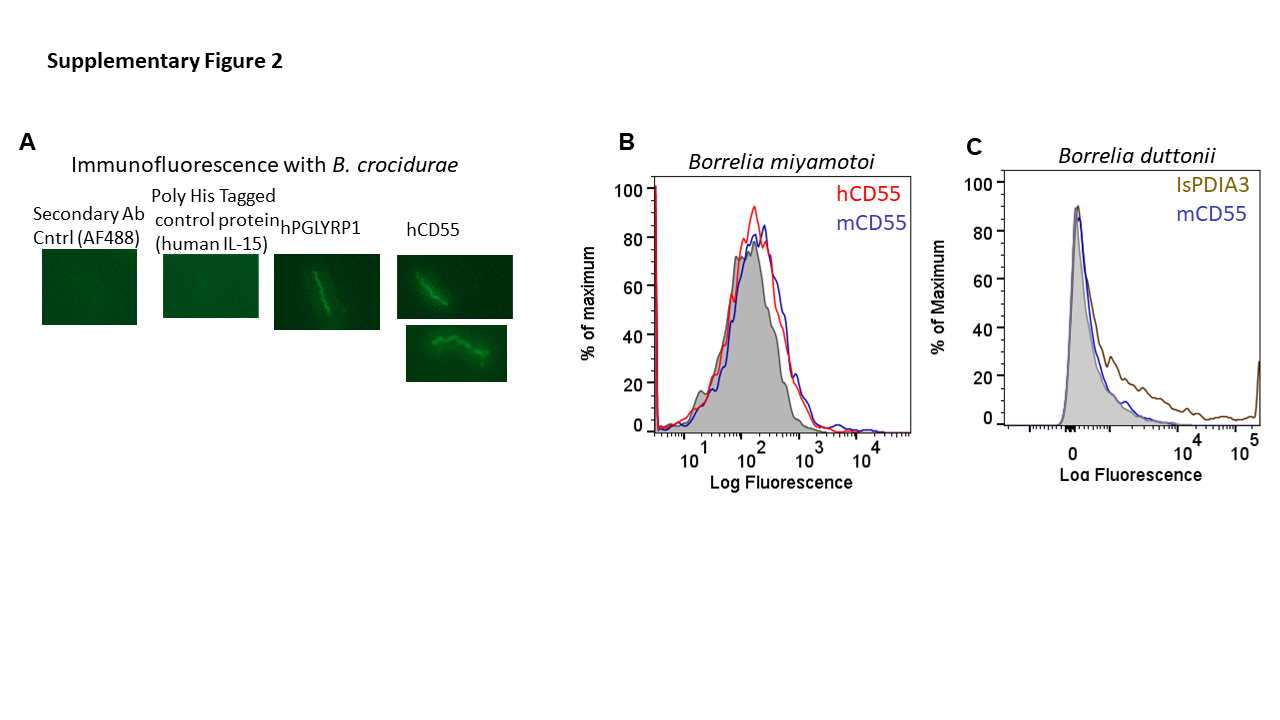

Supplement: FIG S2 [file mbio.01161-22-s0002.tif]

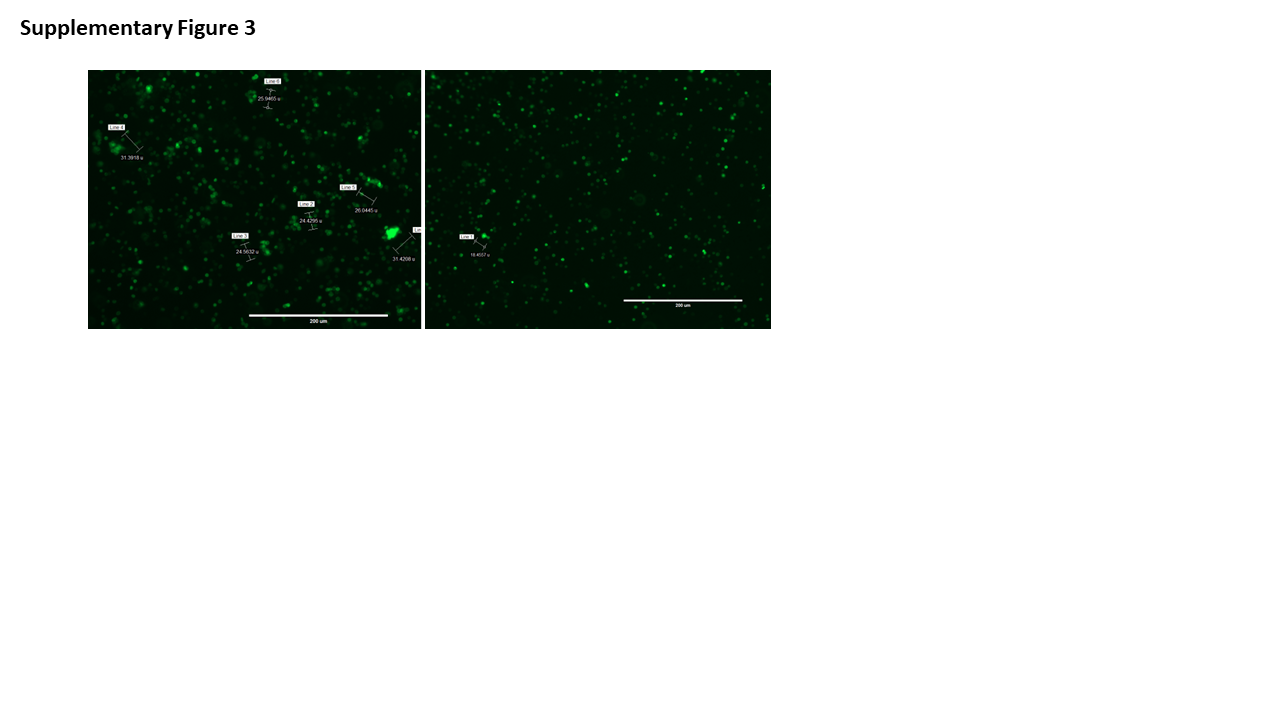

Supplement: FIG S3 [file mbio.01161-22-s0003.tif]

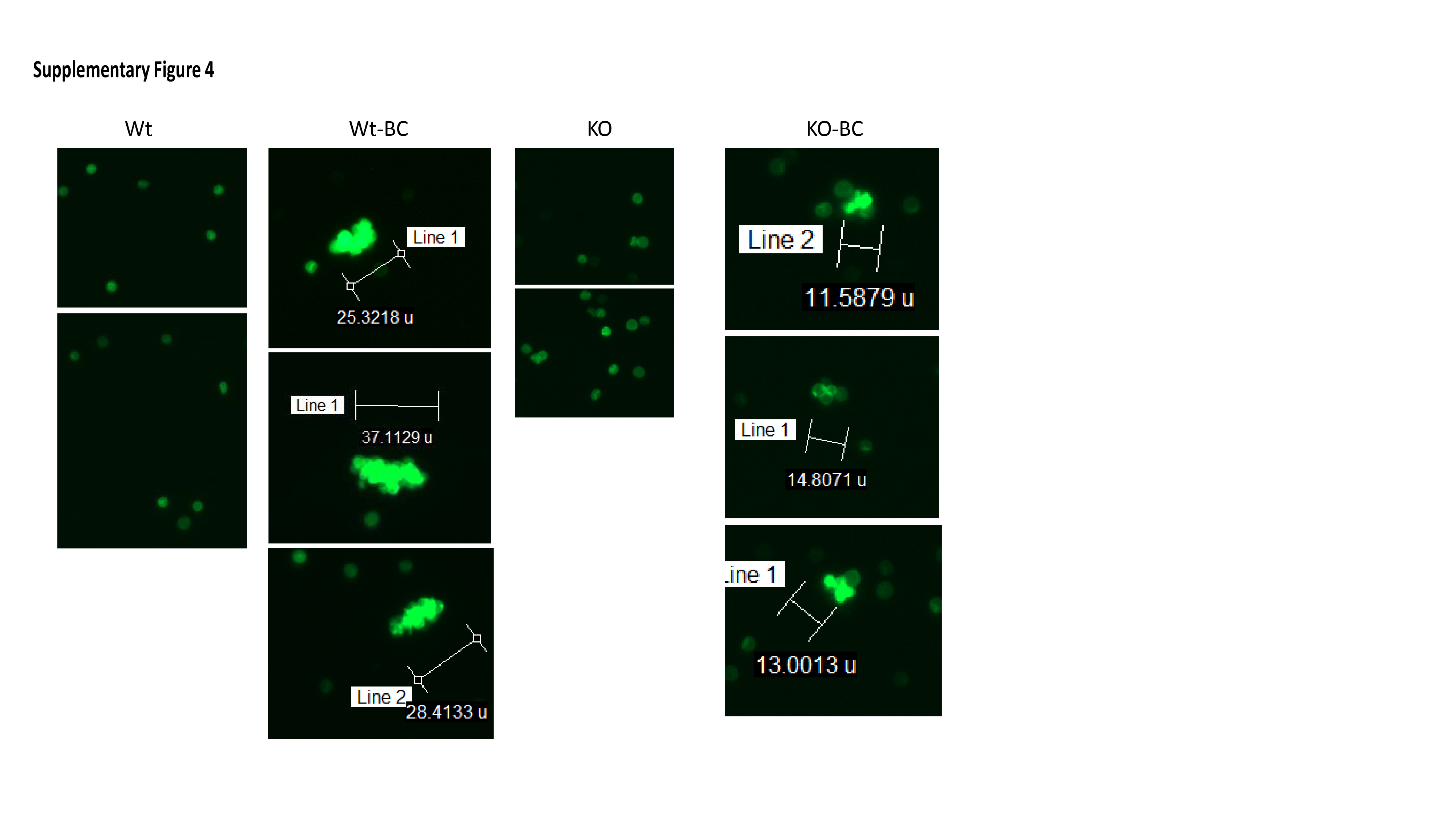

Supplement: FIG S4 [file mbio.01161-22-s0004.tif]

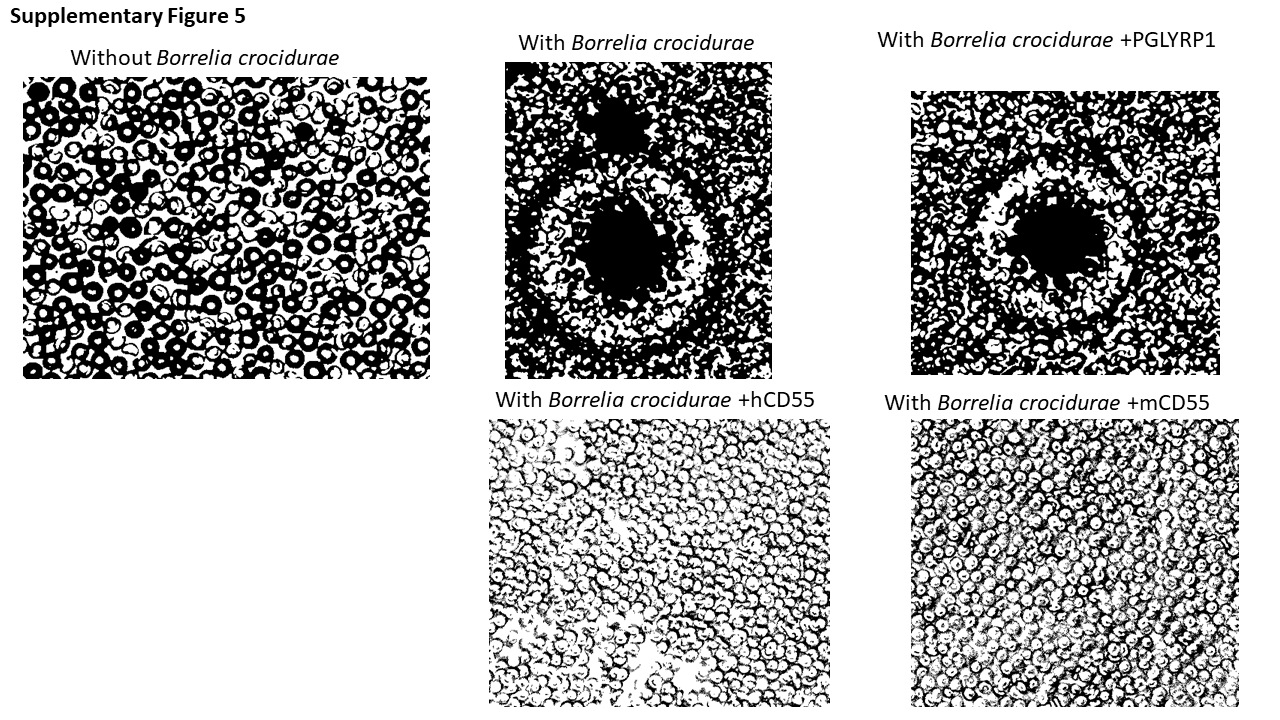

Supplement: FIG S5 [file mbio.01161-22-s0005.tif]

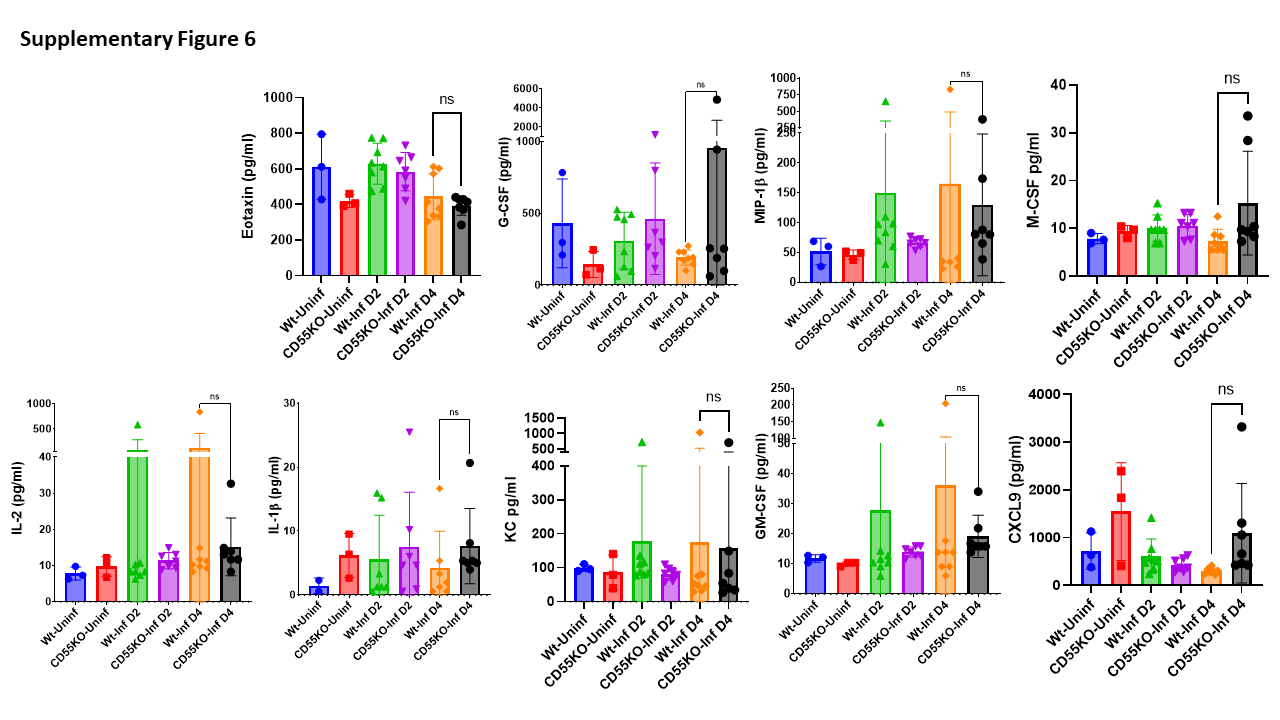

Supplement: FIG S6 [file mbio.01161-22-s0006.tif]

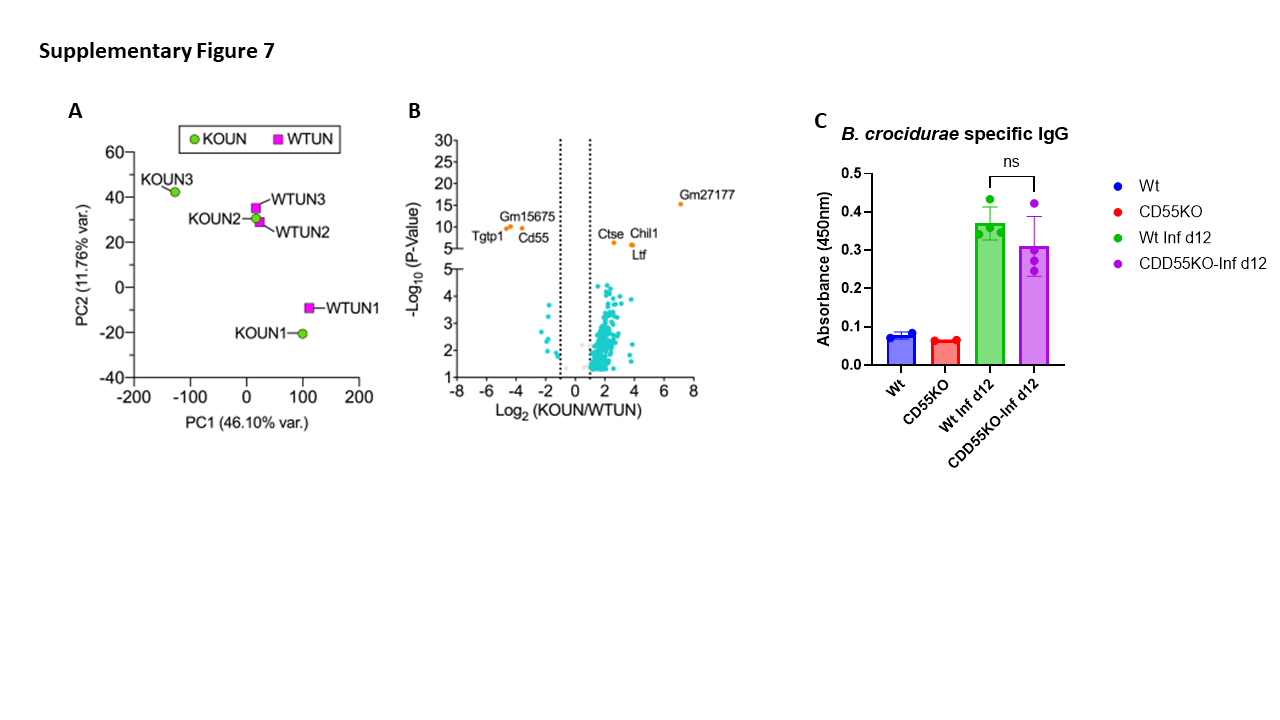

Supplement: FIG S7 [file mbio.01161-22-s0007.tif]
